# Incidence of acute type A aortic dissection in emergency departments

**DOI:** 10.1038/s41598-020-64299-4

**Published:** 2020-05-04

**Authors:** Maximilian Wundram, Volkmar Falk, Jaime-Jürgen Eulert-Grehn, Hermann Herbst, Jana Thurau, Bernd A. Leidel, Eva Göncz, Wolfgang Bauer, Helmut Habazettl, Stephan D. Kurz

**Affiliations:** 1 Deutsches Herzzentrum Berlin, Institute for Anaesthesiology, Augustenburger Platz 1, 13353 Berlin, Germany; 2 Deutsches Herzzentrum Berlin, Department of Cardiothoracic and Vascular Surgery, Augustenburger Platz 1, 13353 Berlin, Germany; 3 Charité – Universitätsmedizin Berlin, corporate member of Freie Universität Berlin, Humboldt-Universität zu Berlin, and Berlin Institute of Health, Department of Cardiovascular Surgery, Augustenburger Platz 1, 13353 Berlin, Germany; 4 DZHK (German Center for Cardiovascular Research), partner site Berlin, 13353 Berlin, Germany; 5 Vivantes Klinikum Neukölln, Department of Pathology, 13407 Berlin, Germany; 6 Charité – Universitätsmedizin Berlin, corporate member of Freie Universität Berlin, Humboldt-Universität zu Berlin, and Berlin Institute of Health, Campus Benjamin Franklin, Department of Emergency Medicine, 12200 Berlin, Germany; 7 Charité – Universitätsmedizin Berlin, corporate member of Freie Universität Berlin, Humboldt-Universität zu Berlin, and Berlin Institute of Health, Campus Virchow-Klinikum, Department of Emergency Medicine, 13353 Berlin, Germany; 8 Charité – Universitätsmedizin Berlin, corporate member of Freie Universität Berlin, Humboldt-Universität zu Berlin, and Berlin Institute of Health, Institute of Physiology, Charitéplatz 1, Berlin, 10117 Germany

**Keywords:** Cardiology, Aortic diseases

## Abstract

Due to the symptoms, patients with acute type A aortic dissection are first seen by the ambulance service and diagnosed at the emergency department. How often an aortic dissection occurs in an emergency department per year has been studied. The incidence in the emergency department may be used as a quality marker of differential diagnostics of acute chest pain. A multi-institutional retrospective study with the municipal Berlin hospital chain Vivantes and its Department of Pathology and the Charité - University Medicine Berlin was performed. From the Berlin Hospital Society, the annual numbers of publicly insured emergency patients were obtained. Between 2006 and 2016, 631 aortic dissections were identified. The total number of patients treated in the emergency departments (n = 12,790,577) was used to calculate the “emergency department incidence.” The autopsy data from six clinics allowed an estimate on how many acute type A aortic dissections remained undetected. Across all Berlin hospitals, the emergency department incidence of acute type A aortic dissection was 5.24 cases in 100,000 patients per year. In tertiary referral hospitals and, particularly, in university hospitals the respective incidences were markedly higher (6.7 and 12.4, respectively). Based on the autopsy results, about 50% of the acute type A aortic dissection may remain undetected, which would double the reported incidences. Among different hospital types the emergency department incidences of acute type A aortic dissection vary between 5.93/100,000 and 24.92/100,000. Aortic dissection; Incidence; Emergency Department; Epidemiology

## Introduction

Acute type A aortic dissection (ATAAD) is part of the preclinical differential diagnosis of acute chest pain. According to the current literature, the population-based incidence of ATAAD is stated between 2.1 and 16.3 per 100,000 persons^1–12^. It is associated with a high mortality ranging from 26% in surgically treated patients to 58% in medically treated patients^13^. ATAAD is associated with a high risk for post-traumatic stress disorder and has negative effects on the patients’ physical and mental health^14^. Aortic dissection usually manifests with sudden thoracic or back pain^13,15^. Increased awareness may further accelerate the diagnostic workup^16^. As the majority of patients with symptoms of an ATAAD are admitted through the emergency department (ED), the emergency staff needs to be aware of the expected incidence of ATAAD. Surgical care of an ATAAD is a time critical event and can only be carried out in specialized clinics^17^. Immediate transportation with a ground-based rescue device to the target hospital is the quickest option and is also necessary from a medical law perspective^18,19^. According to the Federal Statistical office, Berlin has 3,613,495 inhabitants and 83 hospitals. The aim of this study was to gather the current knowledge on the incidence of ATAAD among different populations and to determine the true incidence among patients treated in an ED in the region of Berlin.

## Results

### Emergency department incidence und pathology data

With 12,734,308 ED patients from 2006 to 2016 in Berlin and 631 ATAADs, an ED incidence of 4.96/100,000 was calculated. During the observation period from 2006 to 2016, all these 631 clinical cases of ATAAD were transferred to and treated at the German Heart Center Berlin or the cardiac surgery department of Charité-University Medicine Berlin. Of these, 410 (65%) were men and 221 (35%) were women. The average age was 61.4 ± 13.8 years. The youngest patient was 23 and the oldest patient was 90 years old. The mean body mass index (BMI) was 27.2 ± 4.9 kg/m^2^ and ranged from 16.3 to 55.56 kg/m^2^. In 425 (67.8%) cases, a pre-existing arterial hypertension was detected. Eighty-one (12.8%) patients had coronary artery disease. In 43 (6.8%) patients, type 2 diabetes mellitus was detected. Pre-existing aortic diseases such as aortic aneurysm or ectasia of the aorta were found in 86 (13.6%) of the patients. Fifteen (2.4%) patients had a Stanford type B dissection in their clinical history. One hundred ninety (30.1%) had a history of nicotine abuse, and 32 (5.1%) patients were alcohol addicted. Arteriosclerosis was found in 51 (8.1%) of the patients. A hereditary connective tissue disorder (Marfan or Ehlers-Danlos) was found in 18 (2.9%) patients. 66 (10.5%) patients were known to have hypothyroidism. There were only three (0.5%) cases of pregnancy and five (0.8%) cases of cocaine abuse.

The Charité and the Vivantes clinics play a major role in the care of critically ill patients. We assigned all patients to the respective clinics in Berlin. We divided them into three groups. The first group includes all Charité patients, the ED group patients from Vivantes clinics formed the second group. The third and last group includes all other EDs in Berlin. Detailed data including the distribution of patient characteristics among different types of hospitals are shown in Table 1. The Charité and the Vivantes clinics provided us with their own ED patient numbers. With our data, it was possible to calculate the exact incidence in the ED for the respective observation periods.

**Table 1:** Clinical data of all type A aortic dissections treated in Berlin between 2006–2016.

	Berlin	Charité	Vivantes	Other hospitals
**Basic data**				
Number n (%)	631	171	213	247
male	410 (65.0)	112 (65.5)	142 (66.7)	156 (63.2)
female	221 (35.0)	59 (34.5)	71 (33.3)	91 (36.8)
Age at event, mean±SD in a	61.4 ± 13.8 (23–90)	65 ± 13.9 (23–90)	61 ± 13.4 (28–88)	62 ± 14.1 (23–88)
BMI, mean±SD in kg/m2	27.2 ± 4.9 (16.3–55.56)	26.9 ± 4.6 (18.5–44.2)	26.6 ± 5.6 (17.9–55.6)	26.3 ± 5.6 (16.3–51.9)
**Conditions present n (%)**				
Arterial hypertension	425 (67.4)	103 (60.2)	153 (71.8)	169 (68.4)
Coronary artery disease	81 (12.8)	18 (10.5)	29 (13.6)	34 (13.8)
Diabetes mellitus type 2	43 (6.8)	9 (5.3)	19 (8.9)	15 (6.1)
Aortic disease (ectasia, aneurysm)	86 (13.6)	20 (11.7)	33 (15.8)	33 (13.4)
Type B dissection	15 (2.4)	6 (3.5)	3 (1.4)	6 (2.4)
Nicotine abuse	190 (30.1)	41 (24)	79 (37.1)	70 (28.3)
Alcohol abuse	32 (5.1)	6 (3.5)	17 (8)	9 (3.6)
Arteriosclerosis	51 (8.1)	13 (7.6)	20 (9.4)	18 (7.3)
Hereditary connective tissue disorder	18 (2.9)	5 (2.9)	7 (3.3)	6 (2.4)
Hypothyreosis	66 (10.5)	18 (10.5)	22 (10.3)	26 (10.5)
Pregnancy	3 (0.5)	2 (2.1)	0 (0)	1 (0.4)
Cocaine abuse	5 (0.8)	2 (2.1)	0 (0)	3 (1.2)

The Table shows the Pre-existing medical conditions, average age and gender. The basic data are the mean ± SD with the maximum and the minimum in brackets or absolute numbers with percentages in brackets.

In Table 2 the cases of ATAAD, the population and the incidence are compared for the congruent period from 2010–2016. The first column contains all patients in the Berlin region.

**Table 2:** Emergency Department Incidence between 2010 and 2016.

	Berlin	Charité	Vivantes	Other Hospitals
emergency department patients	8,358,812	1,050,994	2,112,610	5,195,208
observation period	2010–2016			
cases of ATAAD	438	131	153	154
extrapolated cases of ATAAD	876	262	306	308
emergency department incidence ATAAD/100,000 (Confidence interval)	5.24 (5.17–5.29)	12.46 (9.2–15.5)	7.24 (6.31–8.21)	2.96 (2.57–3.34)
extrapolated emergency department incidence ATAAD/100,000 (Confidence interval)	10.48 (9.07–11.44)	24.92 (18.05–31.26)	14.48 (12.31–16.01)	5.93 (5.15–6.68)

The Table 2 shows the population and the cases of ATAAD in the emergency departments of all four groups. The first column contains all patients in the Berlin region. The following columns compare the Charité clinics, the Vivantes clinics and all other hospitals. Incidences were calculated as ATAADs of emergency department cases per 100,000, with the confidence interval in brackets.

The following columns compare the Charité clinics, the Vivantes clinics and all other hospitals in terms of cases, the emergency department incidence and the extrapolated data.The Charité has 131 ATAAD cases and an ED population of 1,050,994. This results in an incidence of 12.46/100,000. At the Vivantes hospitals, 153 cases of ATAAD were found among 2,112,610 ED patients resulting in an ED incidence of 7.24/100,000. The remaining Berlin hospitals had 5,195,208 ED patients and 154 ATAAD cases. The ED incidence was 2.96/100,000. (Table 2).The information of the pathology department of six Vivantes clinics was used to estimate how many ATAADs went undetected. The ATAAD was not known prior to the autopsy. Compared with the data from the German Heart Institute and the Vivantes clinics, this suggests that only one of every two ATAAD cases was detected. The details are shown in Table 3. Extrapolating this assumption to all hospitals, the incidence of ATAAD in the EDs all over Berlin would increase to 10.48/100,000, to 14.48/100,000 at Vivantes, to 24.92/100,000 at the Charité, and to 5.93/100,000 at all other hospitals in Berlin **(**Table 2**)**.

**Table 3:** Pathology Vivantes.

Year	Deceased	Pathology Vivantes	Calculated Autopsy Rate	Cases of ATAAD in Autopsy	Extrapolated Cases of ATAAD in all deaths
2010	5288	253	4.78	0	0
2011	5147	236	4.59	2	44
2012	5397	321	5.95	1	17
2013	5717	402	7.03	7	100
2014	5323	386	7.25	2	28
Total	26872	1598	5.95	12	202

The Table 3 presents the cases in the pathology of the Vivantes clinics. The cases of ATAADs in the autopsies were used to extrapolate the cases of ATAAD in all deceased patients in the Vivantes Clinics. Over the five years, 1,598 autopsies were accomplished. Doing this, 12 additional ATAADs could be found. With a calculated autopsy rate of 5.95 and 26,872 deceased patients in total, 202 cases of ATAAD in all deceased could be extrapolated.

## Discussion

The literature review has shown that the data on the incidence of ATAAD vary substantially. The study characteristics are summarized in Table 4. It is important to provide an accurate estimation of the incidence and also to consider its development over the years. This may serve as a quality indicator for emergency services, statistical offices, public health offices, legal medicine, and other healthcare providers. The total population of the 14 studies included 1,733,937,352 person years. The indicated incidences of all population-based studies were very heterogeneous and ranged from 2.1 to 16.3/100,000 inhabitants^1–12^.

**Table 4:** Incidences of ATAAD as Obtained from Literature Search.

kind of incidence	Author, Date	Type	Population size × observation years	Time period	Incidence per 100.000
population based	Landenhed *et al*. 2015^5^^,a^	P	608,240	1923–1950	15
	Sato *et al*. 2005^6^^,b^	P	1,569,000	1998–1999	4
	Howard et al^7^^,a^.	P	1,020,008	2002–2012	6
	Clouse et al^8^^,a^	R	1,529,122	1980–1994	3.5
	Olsson *et al*. 2006^4^^,a^	R	139,200,000	1987–2002	males: 16.3 females: 9.1
	Melvinsdottir *et al*. 2016^9^^,b^	R	6,381,584	1992–2013	2.53
	Mody et al^10^^,b^.	R	336,781,989	2000–2011	10
	Pacini *et al*. 2013^11^^,b^	R	36,000,000	2000–2008	4.7
	McClure *et al*. 2018^12^^,a^	R	167,070,000	2002–2014	4.6
	Yeh *et al*. 2015^3^^,a^	R	184,383,579	2005–2012	5,6
	Kurz *et al*. 2017^2^	R	29,366,656	2010–2014	11.9
	Reutersberg *et al*. 2019^1^^,b^	R	729,995,776	2006–2014	2.1
emergency department	Rogers *et al*. 2011^20^^,c^	R	100,000,000		10
forensic	Kurz *et al*. 2017^2^	R	﻿12,603	2010–2014	1150.5
autopsy	Mollo, Comino, and Passarino 1983^21^^,c^	R	31,398	1932–1981	595

The Table 4 shows the study characteristics. The Studies are separated by their type of incidence. The second column names the author of the study. The next column includes the study type. The penultimate column presents the population size x observation period. The last column demonstrates the incidence of the acute type A aortic dissection per 100,000.

Explanation:study type: P = prospective study, R = retrospective study.

^a^Includes thoracic aortic dissections (type A and type B), aneurysm and ruptures.

^b^Includes thoracic aortic dissections (type A and type B).

^c^Acute Aortic Dissection without separating the different types.

A retrospective study from Rogers *et al*. 2011 roughly estimated an ED incidence for acute aortic dissection in the United States. They assumed 10,000 cases of AAD annually and 100,000,000 ED visits in the same time period. This led to an ED incidence of 10/100,000 patients^20^.

Our study confirmed this assumption using exact numbers of ED patients and ATAAD cases.

Another study analyzed the incidence in clinical and forensic autopsies in the regions of Berlin and Brandenburg. The analysis provided 1150.5 cases of ATAAD per 100,000 autopsies^2^. In a retrospective study from Mollo *et al*. in 1983, autopsy reports were analyzed from 1932 to 1981. They could show a relative frequency of 1/168 or an incidence of 595/100,000 autopsies^21^. The incidence rates in the population-based studies were related to AADs without distinction between type A and type B. For this reason, we calculated the incidence for the ATAADs from the given data. A population-based incidence of 2.3/100,000 for all population-based studies was obtained. Due to the high preclinical mortality and missing forensic and clinical autopsy data, the number of unreported cases can safely be assumed to be very high. 26% of the patients who received surgical treatment due to a type A dissection died within the clinic. The mortality of the conservatively treated patients was 58%^13^. The ED incidence in the Berlin area varied over the years, but no sustained increase occurred. Across all 83 hospitals in Berlin, an ED incidence of 4.96/100,000 was calculated for the period from 2006 to 2016. The German Heart Center is responsible for the surgical care of nearly 90% of acute type A aortic dissections in the Berlin region. Looking separately at the largest hospital companies, the incidence at the Vivantes clinics was 7.24/100,000, and at the Charité hospitals it was 12.46/100,000 emergency patients. Hospitals with a higher level of care and a simultaneous “Chest Pain Unit” qualification are increasingly being approached by the ambulance service for the symptom of chest pain. With greater expertise and better occupation in the ED, these institutions generate larger numbers of cases and have a higher incidence of ATAAD in the ED. The study showed higher prevalence with lower incidence of ATAAD in the Vivantes clinics compared to the Charité clinics. This is due to the different number of EDs (Vivantes n = 9, Charité n = 3). The autopsy results and these data led to the conclusion that probably only one in two ATAADs was diagnosed. Following this assumption for the time period from 2010 to 2016, the incidence for all Vivantes clinics increased from 7.24/100,000 to 14.48/100,000. Extrapolating this assumption, the incidence in the Charité increased from 12.46/100,000 to 24.92/100,000. There is only one study that assessed the incidence in EDs in the United States of America. It provides a rough estimateof 10 AAD cases of any type among 100,000 ED patients^20^. We calculated an ED incidence of 5.24 ATAAD/100,000 for Berlin. Under the assumption that only one out of two cases are actually detected, the incidence increases to 10.48/100,000, which is almost identical to the United States of America. This incidence is higher than the average extrapolated population-based incidence in Berlin. In relation to the Berlin population during the study period (37,843,130 person years) and 1262 extrapolated cases of ATAAD, the population-based incidence was 3.3/100,000. The examination of the section material shows a very high number of unreported cases. *In all deceased patients undergoing autopsy, the ATAAD was not known prior to the autopsy. Whether or not ATAAD was causal for death is not known, but, considering the high in-hospital mortality of untreated ATAAD we can assume that it was the cause of death in the majority of cases*^22^. To reduce this number, it would be necessary to take a closer look at the deceased patients of each hospital. A study by Modelmog *et al*. could show that the autopsy findings are not in line with the diagnosis given on the death certificate in 45% of the cases^23^. Burton *et al*. from 2003 pointed out that an autopsy can reveal important and unsuspected diagnoses. The discrepancy between the clinical diagnosis and the findings of the autopsy has decreased over time. But, it remains so high that a continuation of the autopsies is indicated^24^.Brinkmann *et al*. provides data on the development of clinicopathologic and forensic autopsy rates between 1994 and 1999. Especially in Berlin, the autopsy rate decreased by 3.9% within 5 years, from 15.3% in 1994 to 11.4% in 1999^25^.

The study periods vary between Charité (2010–2016), Vivantes and Berlin (2006–2016). Unfortunately, it was not possible to gather data on the missing years to get equal study periods. The ED population for 2016 was missing and had to be estimated based on the evolution of patient numbers. As a result, the numbers could be under- or overestimated. The autopsy data was provided only from the Vivantes clinics. The overall autopsy rate was 5.95%. This is not enough to provide reliable numbers on undetected cases of ATAAD. Therefore, the extrapolation of these undetected numbers to all Berlin hospitals should be considered with care. For a more detailed assessment, autopsy data from all hospitals in Berlin and the Charité would be necessary. The new data on incidence in EDs should sensitize the hospitals and ambulance services to this acute disease. The goal in preclinical care should be the establishment of a standard operating procedure for the symptom complex of chest pain in which the aortic dissection detection risk score is firmly anchored. There, a special focus should be on patients who are delivered by the ambulance or the rescue service.

## Conclusion

Our results underline a higher incidence in the EDs compared with the population-based incidence in the general population of Berlin. The ED incidence in the period from 2010 to 2016 varies between 2.96/100,000 and 12.46/100,000 among different types of hospitals. The population-based incidence for the entire Berlin population from 2010 to 2016 is 3.45/100,000. Specialized clinics show significantly higher case numbers. The ATAAD is a severe disease and it is one of the four differential diagnoses of acute chest pain. Lower than expected incidences may mean that ATAAD remains undetected in a considerable number of patients. The pathology department information showed, that only one in two cases of ATAAD is detected. Under this assumption, the ED incidences and the population-based incidence would be twice as large.

## Material and Methods

### Study design

The databases PubMed und Google Scholar were used to search for studies on the subject of incidence of aortic dissection. Incidences in the fields of population, ED, pathology autopsies, and forensic medicine were considered. “Acute aortic dissection type A incidence” and “acute aortic dissection at autopsy” were used as search terms. The search on Pubmed and Google Scholar yielded 23,056 studies. The abstracts were screened, and studies were excluded if they dealt with other topics, full-text was not available, or language was different than English or German. Based on these criteria, 23,020 studies were excluded. Twenty-two of the remaining 34 full-text studies fulfilled all criteria for eligibility and met all criteria. The bibliographies of the included studies were explored for further relevant studies. In an additional search, six more relevant studies were identified. Fourteen articles that deal with autopsy-, population-, forensic medicine incidence, and ED incidence of ATAAD are summarized in Table 4, which includes the following information: kind of incidence, author, date, study type, population size and incidence. The study from Kurz *et al*. was considered twice, for a population based incidence and a forensic medicine incidence of ATAAD^2^.

### Data collection

The only two institutions surgically treating ATAAD in Berlin are the German heart Centre and the Department of Cardiovascular Surgery, Charité- University Medicine Berlin. Thus the number of 631 patients was taken from the archives of these hospitals covering the years 2006 to 2016. To estimate a population of ED patients in Berlin for the years 2006 to 2016, we contacted the two major health care providers, the municipal Berlin hospital chain Vivantes, running nine large hospitals, and the Charité-University Medicine Berlin, with three hospital sites. For Charité-University Medicine hospitals, we received the numbers of ED patients for the years 2010 to 2016 directly from the heads of the respective departments. The Vivantes clinics provided us with the data from 2006 to 2015. The missing data from 2016 had to be calculated based on the annual increase. From the Berlin Hospital Society, we received the annual numbers of publicly insured emergency patients for the years 1998 to 2015. Estimating the rate of privately insured patients at 10%, we added the respective numbers. For the year 2016, no data were available. We therefore extrapolated the number for this year according to the average yearly increment of patient numbers, resulting in a total of 1,268,507 patients in 2016, including private patients.

### Ethics approval and consent to participate

The study was approved by the Ethics Committee of Charité-Universitätsmedizin Berlin (Ethics Subcommittee 2 of Campus Virchow-Klinikum, registration number: EA2/126/14) and complies with the Declaration of Helsinki. An informed consent by the patient to participate in the study was not necessary on the part of the ethics committee.

## Data Availability

The datasets used and analyzed during the current study are available from the corresponding author on reasonable request.
